# The mouse posterior insular cortex encodes expressive and receptive aspects of courtship vocalizations

**DOI:** 10.1016/j.celrep.2025.115850

**Published:** 2025-06-14

**Authors:** Thomas Pomberger, Katherine S. Kaplan, Rene Carter, Autumn Wetsel, Thomas C. Harmon, Richard Mooney

**Affiliations:** 1 Department of Neurobiology, Duke University, Durham, NC 27710, USA; 2 Lead contact

## Abstract

Socially effective vocal communication requires brain regions that encode expressive and receptive aspects of vocal communication in a social context-dependent manner. Here, we combined a novel behavioral assay with microendoscopic calcium imaging to interrogate neuronal activity (regions of interest [ROIs]) in the posterior insula (pIns) in socially interacting mice as they switched rapidly between states of vocal expression and reception. We found that largely distinct subsets of pIns ROIs were active during vocal expression and reception. Notably, pIns activity during vocal expression increased prior to vocal onset and was also detected in congenitally deaf mice, pointing to a motor signal. Furthermore, receptive pIns activity was modulated strongly by social context. Lastly, tracing experiments reveal that deep-layer neurons in the pIns directly bridge the auditory thalamus to a midbrain vocal gating region. Therefore, the pIns is a site that encodes vocal expression and reception in a manner that depends on social context.

## In brief

Pomberger et al. show that the mouse posterior insula encodes vocal expression and reception in a socially sensitive manner. Largely distinct neuronal populations respond during vocal production and listening, with modulation shaped by social context and sensory cues. Anatomical tracing links the insula to key auditory and vocal motor structures.

## Graphical abstract

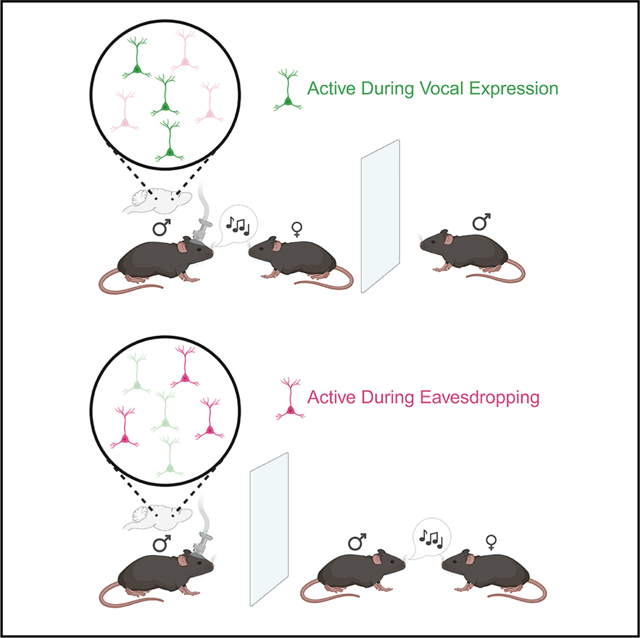

## INTRODUCTION

Vocal communication is an essential medium for forging and maintaining social bonds in all mammalian species, including humans.^1–4^ Socially effective vocal communication requires that vocal expression and reception (i.e., listening) are carefully regulated as a function of social context. For example, vocalizations require an audience to exert their social effects, and the audience must in turn discern which vocalizations signify socially relevant exchanges. A major unresolved issue is the extent to which single brain regions encode expressive and receptive aspects of vocal communication in a manner that is sensitive to social context.

The insular cortex binds various sensory and social signals to guide behavior,^5–11^ providing a potential site for encoding socially salient vocal signals. In fact, the posterior insula (pIns) integrates multisensory information, including auditory stimuli, in monkeys^12^ and mice.^13–15^ In monkeys, pIns neurons respond to a range of animal vocalizations, with the strongest responses evoked by conspecific vocalizations.^12^ Although pIns activity during vocal expression has yet to be described in monkeys or rodents, intracranial electroencephalography (iEEG) recordings in human subjects show enhanced activity during speech as well as speech playback,^16^ and human patients with insula lesions suffer from articulatory planning deficits.^17,18^ Therefore, the pIns is an attractive candidate brain region where expressive and receptive aspects of vocal communication may be encoded in a manner that is sensitive to social context.

Our understanding of how the pIns encodes expressive and receptive aspects of vocal communication is currently limited. First, most studies of the auditory properties of pIns neurons have presented vocalizations through a speaker to head-fixed, socially isolated animals, a state where vocalizations are devoid of social context. Furthermore, a systematic characterization of the same populations of pIns neurons during social interactions that involve both vocal expression and reception has yet to be undertaken. While the recent characterization of the pIns in humans is a step in this direction, iEEG lacks cellular resolution, and how social context modulates pIns activity remains unknown.

Here, we combined a novel behavioral assay with miniature microendoscopy (miniature microscope [miniscope]) in which we could interrogate pIns neuronal activity in socially interacting mice as they switched rapidly between states of vocal expression and reception. We found that distinct but spatially intermingled subsets of pIns neurons were active during these two states. Notably, pIns activity during vocal expression increased prior to vocal onset and was also detected in congenitally deaf mice, consistent with a motor-related signal. Moreover, the subset of pIns neurons that were activated when a mouse listened to vocalizations produced during social encounters was activated only weakly or not at all by vocal playback when the mouse was by itself. Further analysis of pIns activity using multiphoton imaging in head-fixed male mice in which we could carefully regulate exposure to a female mouse revealed that female odorants enhanced pIns responses to vocal playback. Lastly, tracing experiments reveal that deep-layer neurons in the pIns directly bridge the auditory thalamus to a vocal gating region in the periaqueductal gray (PAG). These findings identify the pIns as a site where auditory and motor representations of vocal communication signals are represented in a manner that depends on social context.

## RESULTS

### A behavioral protocol for monitoring social-vocal communication

Male mice emit ultrasonic vocalizations (USVs) when exposed to female mice or their odors,^19–22^ and these vocalizations facilitate mating.^23,24^ This courtship behavior provides an ethologically relevant context in which to explore the neural correlates of expressive (in the male) and receptive (in the female) aspects of social communication. Furthermore, while the male emits courtship USVs in response to a female mouse or associated female odorants, in natural settings, other mice, including rival males, can eavesdrop on these vocal bouts and thus detect these courtship encounters. Therefore, eavesdropping males provide an additional context in which to probe the neural correlates of vocal reception.

**Figure 1.**
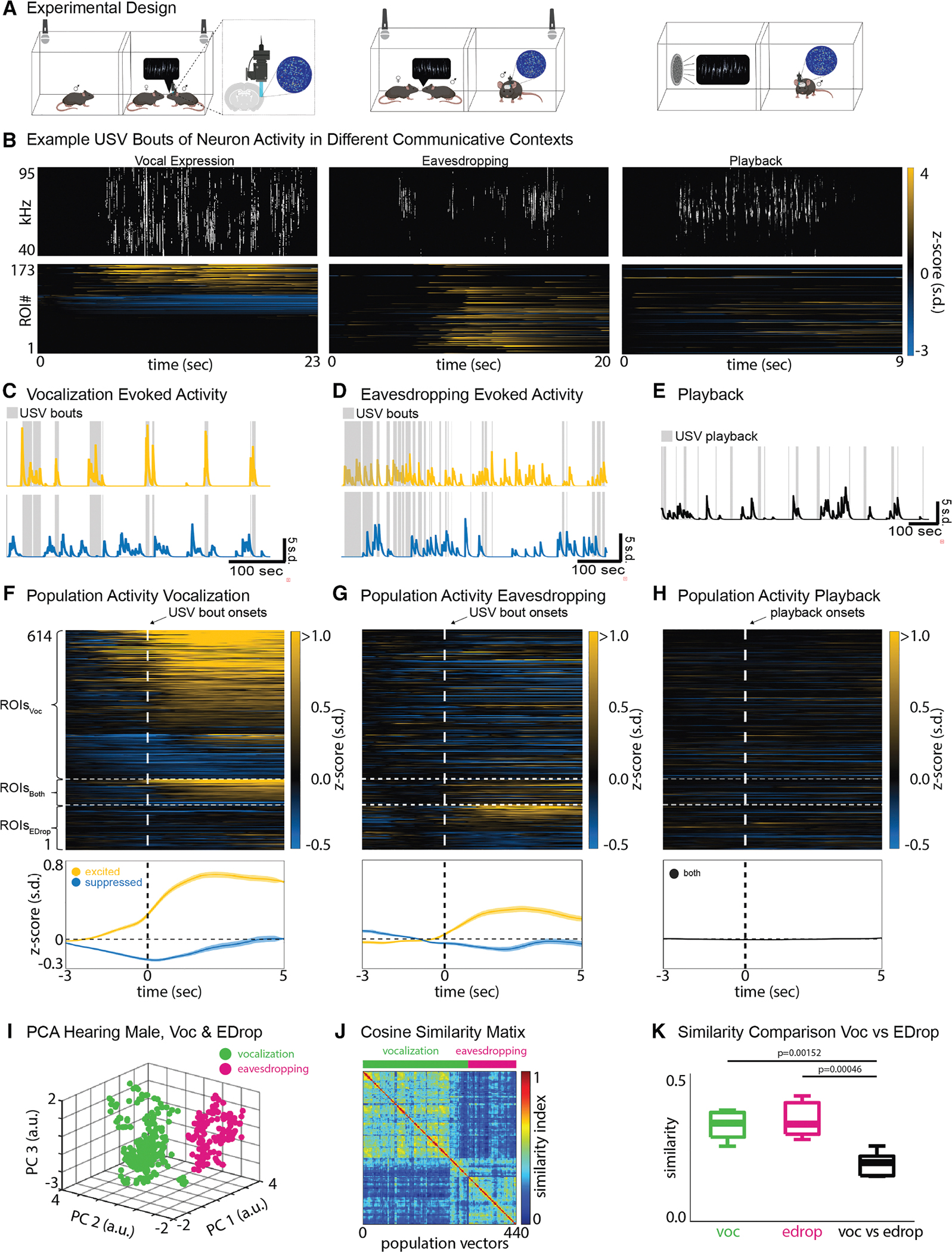
The posterior insula is active during socially salient vocal expression and reception (A) Experimental design showing the three different social-vocal contexts. (B) Example USVs during vocal expression (top left), eavesdropping (top middle), and playback (top right), and the corresponding ROI activities below (mean ± SEM). (C–E) Example ROIs showing activity during USV bouts and USV playback for each of the three communicative contexts (yellow = excited, blue = suppressed, and black = non-responsive). (F and G) Trial average activity of ROIs (*Z* scored, *N* = 5 males) that are active during vocal expression (ROIs_Voc_), during eavesdropping (ROIs_EDrop_), or in both contexts (ROIs_Both_). The top images show each individual ROI. The bottom images show the overall population activity (mean ± SEM) of excited (yellow) and suppressed (blue) ROIs. (H) Same ROIs as in (F) and (G) but shown during USV playback. (I) Principal-component analysis (PCA) of population vectors from a representative hearing male that shows that co-active groups of ROIs during vocalization (green) form a different cluster than those co-active during eavesdropping (magenta). Each dot represents a population vector. (J) Sorted similarity map of the male from (I) showing the lack of overlap between population vectors in vocalization and eavesdropping. (K) Cosine similarities of population vectors related to vocalization and eavesdropping are significantly different (*N* = 5 males, Kruskal-Wallis, *p* < 0.05).

In order to study the neural correlates of these expressive and receptive processes, we developed a two-chamber system in which to probe neural activity in the pIns in male and female mice during social encounters in which the males typically emit USVs (Figure 1A). In this setup, a male mouse was housed with a female in a ‘‘courtship’’ chamber while another male was placed in an adjacent ‘‘eavesdropping’’ chamber. The two chambers were separated by a mesh screen through which auditory signals and odors were transmitted. The movements of the individual mice were monitored under infrared illumination, eliminating any social signals provided by visual cues. Microphones over each chamber were used to detect USVs and establish that vocalizations emanated exclusively from the courtship chamber. We assumed that the majority of these USVs were produced by the male in the courtship chamber, given that female mice rarely emit USVs during courtship encounters.^25–27^ Therefore, both the female and the male in the adjacent chamber served as receivers for the courting male’s USVs. By moving the female from one chamber to the other, we were able to switch a male’s role from emitting USVs during courtship to eavesdropping on the other male’s courtship USVs. Finally, we isolated the experimental mouse and delivered a series of pre-recorded male USVs through a speaker, allowing us to measure auditory responses to vocalizations in the absence of social cues.

### The pIns is active during socially salient vocal expression and reception

We combined our behavioral approach with calcium imaging using a miniscope to monitor pIns activity during expressive and receptive phases of socially salient vocal communication and when vocal stimuli were presented in social isolation. Briefly, we used adeno-associated viral vectors (AAV2/9-hSyn-GCaMP8s-WPRE) to express GCaMP8s pan-neuronally in the pIns and a gradient index (GRIN) lens to gain optical access to superficial and deep layers of this region (Figures 1A, left, and S1). We used the automated analysis pipeline minian^28^ to extract regions of interest (ROIs) for further analysis. Qualitatively, the activity of the pIns increased sharply in male mice when they emitted USVs during courtship interactions with a female and when they eavesdropped on live USV bouts of another male suitor (Figure 1B, left and middle). In contrast, pIns ROIs were only weakly activated in trials where socially isolated males listened to USV bouts played through a speaker (Figure 1B, right).

To more systematically quantify these effects, we performed a receiver operating characteristic (ROC) analysis, allowing us to identify subsets of ROIs that were significantly excited or suppressed relative to baseline during USV production, eavesdropping, and playback (Figure S2). This approach confirmed that subsets of ROIs in the pIns were significantly excited or suppressed during vocal production and eavesdropping (Figures 1C and 1D, left top and bottom) but not during playback (Figure 1E). Furthermore, the ROC analysis revealed that the population of pIns ROIs active during self-produced vocalizations (pIns_Voc_) was mostly non-overlapping with the population active during eavesdropping (pIns_EDrop_) (Figures 1F and 1G, right top and bottom). Furthermore, population vector analysis revealed a clear separation between pIns subpopulations that were active during vocal expression from eavesdropping activity (Figures 1I–1K). A smaller number of ROIs were modulated during self-produced vocalizations and eavesdropping (Figures 1F and 1G, right middle). We did not observe substantial differences in the proportions of ROIs exhibiting high temporal variability across the different contexts (Figures S3A–S3C). Therefore, largely distinct populations of ROIs in the pIns are activated with similar temporal profiles during expressive and receptive aspects of vocal communication.

In contrast, pIns ROIs were unresponsive to non-vocal noises that accompanied social encounters, such as cage noises (Figure S4A), and were only very weakly activated when socially isolated mice listened to the same vocalizations played through a speaker (Figure 1H). In total, ~22% (466 of 1,992) of ROIs in the pIns were significantly modulated from baseline during self-initiated vocalizations, and ~10% (209 of 1,992) were modulated during eavesdropping (Figure S5G, *N* = 5 male mice, Wilcoxon, *p* < 0.05). Furthermore, ~9% of all responsive ROIs (61 of 675) were significantly modulated from baseline during both vocal production and eavesdropping (Figure S5H). In contrast, only 1 ROI was significantly modulated by USV playback. Thus, auditory responses in the pIns are preferentially evoked by USVs and are sensitive to the social context in which they are heard.

A notable feature of the population of pIns_Voc_ ROIs was that their activity deviated from baseline prior to vocal onset, whereas activity in the pIns_EDrop_ population deviated after the onset of the other male’s USVs (Figures 1F and 1G, bottom, S3A, and S3B). Therefore, modulation of pIns activity during vocal production was not purely a consequence of vocalization-related auditory feedback and instead may reflect a premotor signal. We also examined whether these two populations of pIns ROIs were spatially distinct. Qualitatively, pIns_Voc_ and pIns_EDrop_ appeared to be intermingled across the imaging field of view (Figures S5G and S5H, top). Furthermore, the probabilities of pairwise Euclidean distances between pIns_Voc_ and pIns_EDrop_ ROIs were closely overlapping, indicating these two populations have similar spatial distributions in the insula (Figure S5I; two-sided Kolmogorov-Smirnov [KS] test, *p* = 0.71). In summary, largely distinct populations of spatially intermingled pIns ROIs are active during vocal expression and reception in male mice and may separately encode motor versus auditory information about USVs.

### Activity during vocal expression is not attributable to locomotion

**Figure 2.**
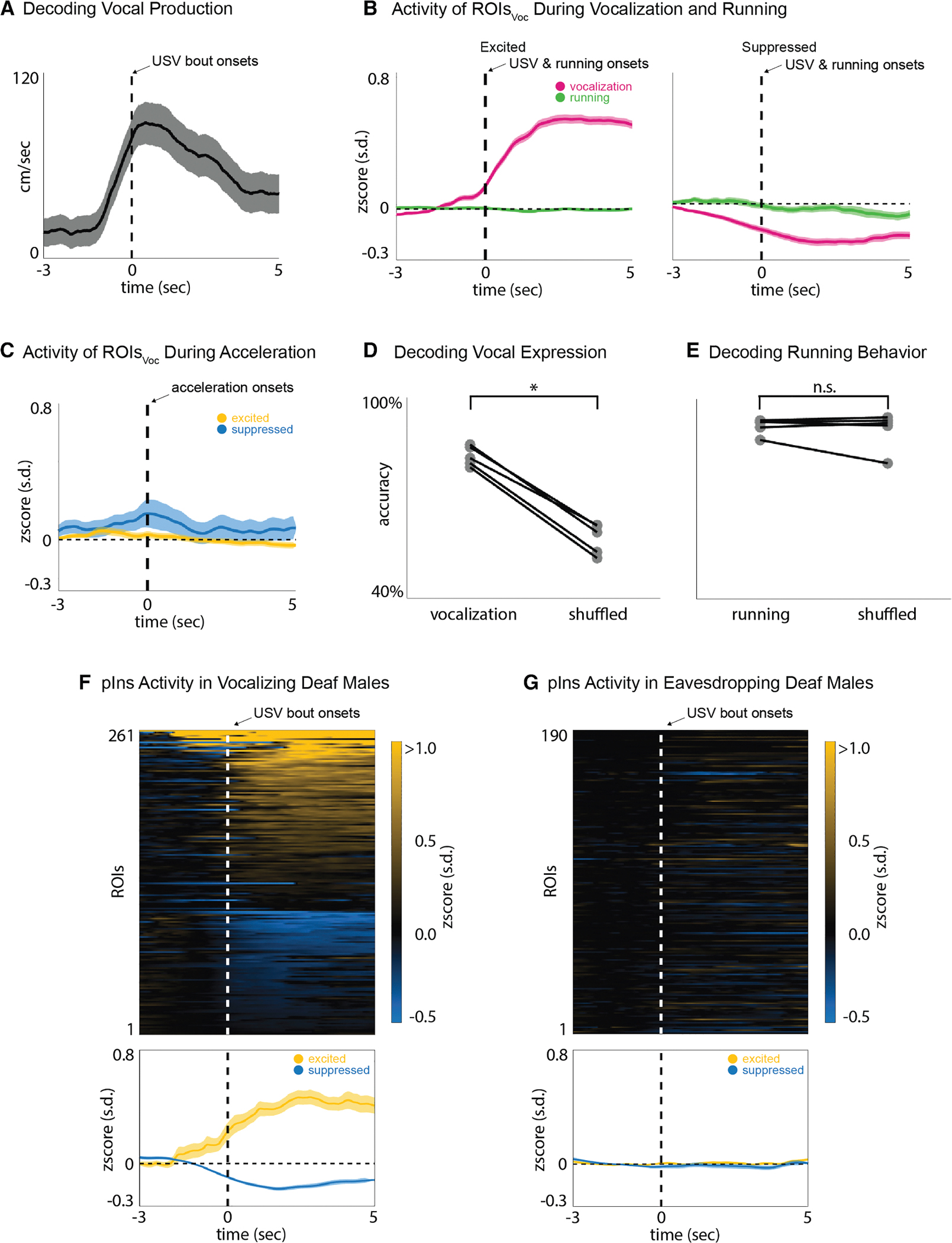
Activity during vocal expression is not attributable to locomotion and does not require hearing (A) Average running speed of vocalizing male aligned to USV bout onset (mean ± SEM, *N* = 5 males). (B) Average population activity (mean ± SEM, *N* = 5 males) of excited (left) and suppressed (right) ROIs_Voc_ aligned to USV bout onset (magenta) and running bout onset (green). (C) Average population activity of excited (yellow) and suppressed (blue) ROIs_Voc_ aligned to acceleration bout onset (mean ± SEM, *N* = 5 males). (D and E) Decoding accuracies for vocal expression (right, *N* = 5, Wilcoxon, *p* < 0.05) and running (left, *N* = 5, Wilcoxon, *p* = 0.69). (F) Trial average activity of ROIs (*Z* scored, *N* = 5 males) that are active during vocal expression in deaf males (top) and the corresponding average population activity of excited (yellow) and suppressed (blue) ROIs (mean ± SEM). (G) Same as in (F) but for activity during eavesdropping in deaf males (*N* = 4 males).

Vocalization in freely behaving male mice typically occurs during female pursuit, and locomotion can modulate activity in sensory cortices, including the auditory cortex (AuC).^29–31^ In fact, we confirmed that the male’s running speed increased prior to USV onset, raising the potential confound that vocal modulation of pIns activity was driven by locomotion rather than vocal production (Figure 2A). However, aligning pIns_Voc_ ROIs to either running onset or acceleration in running speed failed to detect any change in fluorescence (Figures 2B and 2C, Wilcoxon, *p* = 0.31). Furthermore, we trained a long short-term memory (LSTM) network to decode either vocalization or running from the entire population of pIns ROIs (1,992 ROIs from *N* = 5 male mice). The decoding accuracy of the LSTM was significantly greater for vocalization when compared to shuffled data, while the running state could not be decoded (Figures 2D and 2E; Wilcoxon rank sum, *p* < 0.05 and *p* = 0.69). Therefore, activity in pIns_Voc_ ROIs is a consequence of vocal production rather than locomotion.

Because male USVs are produced as the male pursues and approaches the female, another possibility is that social approach, rather than vocalization, is the primary source of modulation of pIns_Voc_ ROIs. To test this idea, we analyzed the activity of pIns_Voc_ ROIs during approach bouts in which no USVs were produced. We found that ~75% of these ROIs were inactive during such non-vocal social approaches (Figures S4B and S4C). These findings further support the conclusion that pIns activity is more specifically associated with USV expression than the social approach behavior that accompanies USV production.

### Activity during vocal expression does not require hearing

As previously noted, activity in pIns_Voc_ ROIs increased prior to vocal onset, indicating that activity during vocal expression is not limited to vocalization-related auditory feedback. To further probe the extent to which vocal modulation in pIns_Voc_ ROIs was independent of auditory feedback, we monitored pIns activity in congenitally deaf males as they engaged in courtship or eavesdropping (Tmc1(Δ)).^32^ In a previous study, we established that adult Tmc1(Δ) mice lack an auditory brainstem response (ABR) and emit a USV repertoire indistinguishable from hemizygous, hearing littermates.^31^ Here, we genotyped each Tmc1(Δ) male and performed ABR tests in a subset (3 of 5 animals) to confirm that they could not hear (Figure S3H).

Notably, calcium signals in a subset of pIns ROIs in Tmc1(Δ) male mice were modulated from baseline during vocal expression (Figures 2F and S3D). The proportions of vocalization-modulated pIns_Voc_ ROIs (~25%, 261 of 1,063 ROIs, *N* = 5 males) and the time courses of their vocal modulation were similar in deaf and hearing mice (Figure S3I, Wilcoxon rank sum, *p* = 0.55 for excited and *p* = 0.22 for suppressed ROIs). One difference was that cosine similarities of pre-vocalization and vocalization phases were higher in Tmc1(Δ) males, suggesting that vocalization-related activity in the pIns of males was less variable in the absence of hearing (Figures S5A–S5F). Furthermore, no modulation occurred when Tmc1(Δ) males were placed in the eavesdropping chamber and exposed to another male’s courtship USVs (Figures 2G and S3E, *N* = 4 males), confirming that eavesdropping-related activity depends on hearing. Therefore, modulation of pIns activity during vocal expression does not require auditory feedback, pointing to the presence of either a motor or a proprioceptive signal.

### pIns ROIs in the female mouse respond to socially salient USVs

**Figure 3.**
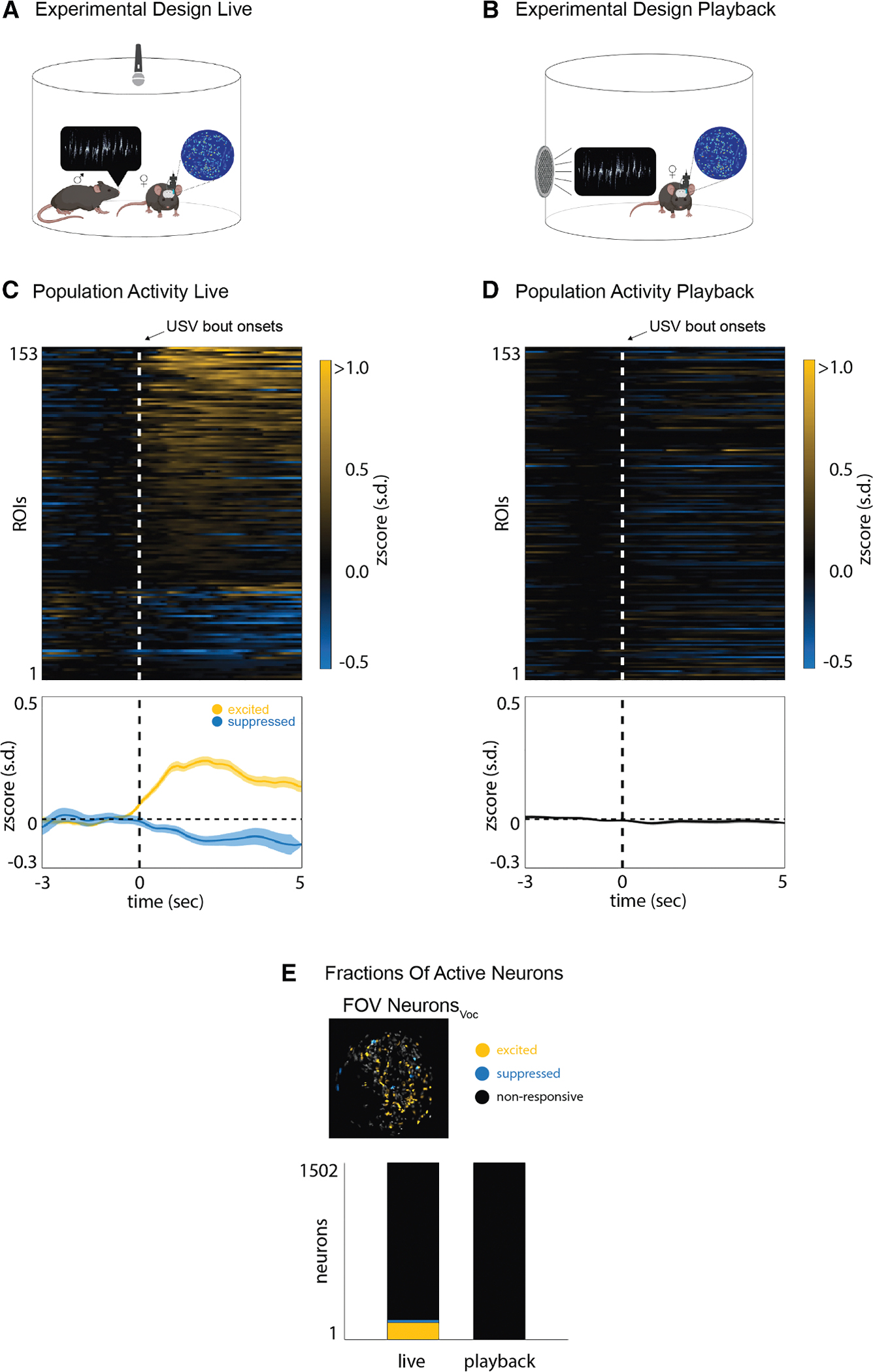
pIns ROIs in the female mouse respond to socially salient USVs (A and B) Experimental design for live and playback vocalizations. (C) Trial average ROI activity in the pIns of females (*Z* scored, *N* = 5) that were exposed to a vocalizing male (top). The bottom image shows the average population activity of excited (yellow) and suppressed (blue) ROIs (mean ± SEM). (D) Same ROIs as in (C) but shown during USV playback. (E) Example field of view of excited (yellow) and suppressed (blue) ROIs of a female when exposed to a vocalizing male (top). The total amount of ROIs for each of the two contexts (live vocalization and playback) is shown.

We also imaged pIns ROIs in female mice housed in the courtship chamber with a vocalizing male (Figure 3A). A subset of pIns ROIs in females responded strongly to USVs of a male suitor (Figures 3C and S3F). An ROC analysis quantified 153 of 1,500 ROIs as USV responsive, similar to the proportion of USV-responsive pIns ROIs detected in eavesdropping males (Figure 3E, *N* = 5 females). As in eavesdropping males, the responses of female pIns ROIs to male USVs were strongly dependent on social context, as no ROIs were modulated by USV playback when these females were in social isolation (Figures 3D and S3G). In summary, a subset of pIns ROIs are active during vocal reception in both female and male mice.

### Female odor increases auditory responsiveness in male pIns

**Figure 4.**
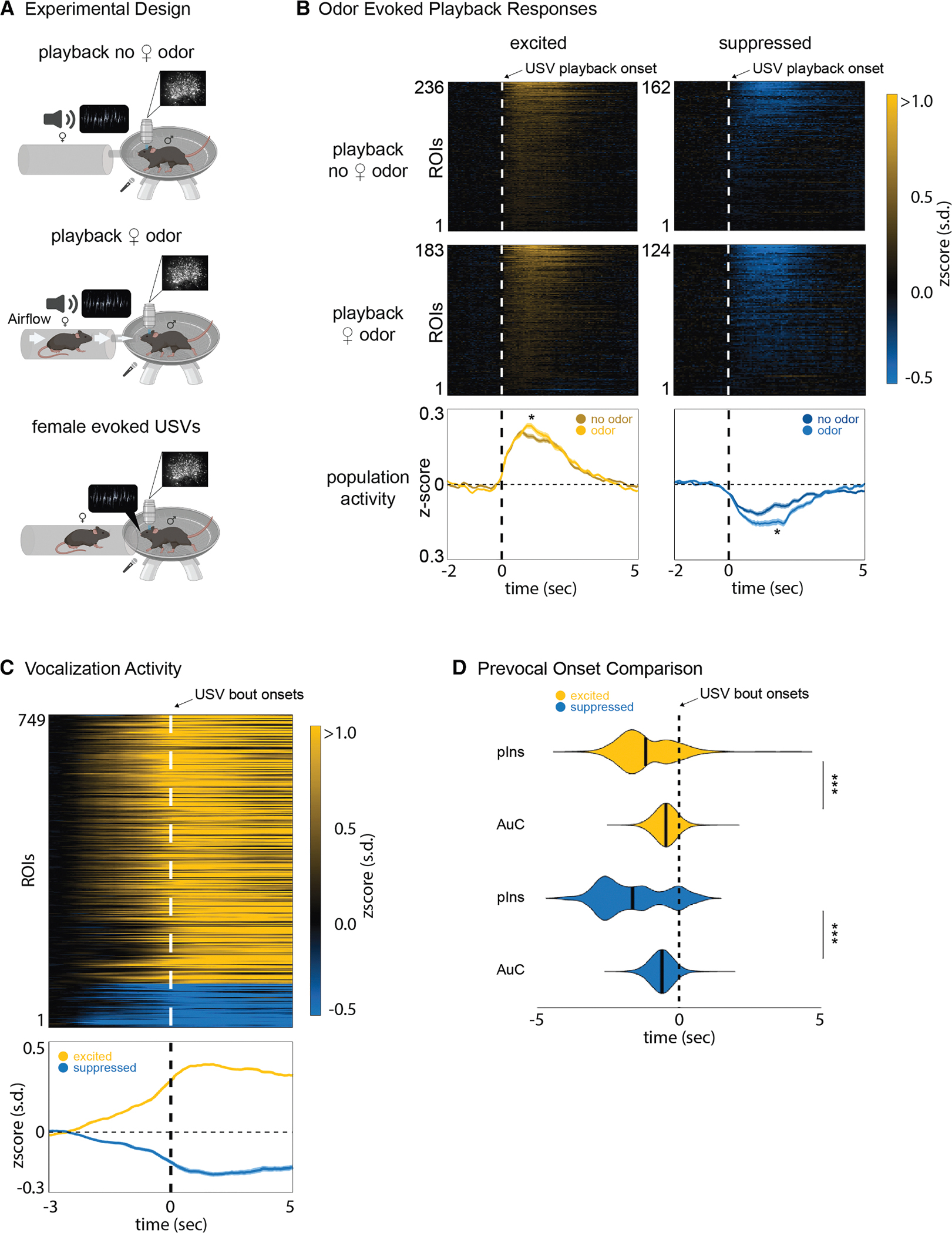
Female odor increases auditory responsiveness in male posterior insula (A) Experimental design showing the head-fixed male exposed to USV playback during neutral airflow (top), positive airflow that delivers odorants from a distal female (middle), and USVs elicited by an approaching female (bottom). (B) Average activity of ROIs (*N* = 4 males, *Z* scored) that were active during neutral and positive airflow (top and middle). The bottom image shows the average population activity of excited (yellow) and suppressed (blue) ROIs during neutral and positive airflow (mean ± SEM, *N* = 4 males). Stars indicate significance between the two populations (Wilcoxon, *p* < 0.05). (C) Average activity of ROIs that were active during vocal expression in head-fixed males. Bottom image shows average population activity of excited (yellow) and suppressed (blue) ROIs. (D) Pre-vocal onset activity of posterior insula (pIns) and auditory cortex (AuC) of excited and suppressed ROI populations.

Here, we found that a subset of ROIs in the pIns of eavesdropping male mice respond strongly to USVs produced by a nearby courting male, whereas USV playback elicits only weak responses in the pIns when males are in social isolation. Therefore, additional non-vocal social cues must augment the responses of pIns neurons to the other male’s USVs. Given the multisensory nature of the pIns,^14,33^ we hypothesized that female odor is one of these social cues. Regulating odor delivery in unrestrained courting mice is impractical, so we instead used 2p methods to image pIns activity in the head-fixed male mouse while regulating its exposure to female mouse odors and delivering pre-recorded USVs of other males through a speaker. In this setting, odors were delivered to the head-fixed male by directing airflow into a chamber containing the female and through a nozzle in front of the male’s snout (Figure 4A, middle). Under conditions of no directed airflow or when the female was absent (Figure 4A, top), a subset of pIns ROIs were either excited or suppressed by USV playback (Figure 4B, top and bottom). The proportion of playback-responsive ROIs using 2p imaging methods (~37%, 499 of 1,351 ROIs) contrasts with the absence of USV playback-excited ROIs detected using miniscopes in socially isolated male mice (Wilcoxon rank sum, *p* < 0.05). When airflow was directed toward the head-fixed male, the magnitude of the excitatory and suppressive responses of these ROIs to USV playback increased (Figures 4B, middle and bottom, *N* = 4 males, Wilcoxon, *p* < 0.05, and S6A, Pearson correlation, *p* < 0.05). Separate from playback responses, we did not detect any differences in fluorescence between the undirected (i.e., no odor) and directed airflow conditions (Figure S6B, two-sided KS test, *p* = 0.17). Therefore, female odorants modulate auditory responses in the male’s pIns to other male’s USVs.

We also conducted an additional set of experiments in which a female mouse could approach the head-fixed male mouse snout to snout, which often elicited USVs from the male (Figure 4A, bottom). As in the miniscope experiments, pIns activity was strongly modulated in the pIns of vocalizing, head-fixed males, and this vocalization-related activity increased before vocal onset (Figure 4C). In fact, similar to the enhanced responsiveness to USV playback, an even greater proportion of pIns ROIs were modulated during USV production under the 2p compared to miniscope imaging (~55%, 749 of 1,351 ROIs). The increased numbers of vocalization- and playback-responsive ROIs detected using a 2p microscope most likely reflect the heightened sensitivity of the 2p method, as the total number of ROIs detected for each mouse was higher in the 2p compared to miniscope conditions (mean 2p: 338 ± 50, mean miniscope: 279 ± 15).

Along with the increased numbers of pIns ROIs activated during vocalization- and playback-responsive neurons detected using 2p methods, we also observed an increased proportion of pIns ROIs that were modulated during vocalization and by USV playback in either the presence or absence of female odor (~33% overlap of all responsive ROIs versus ~9% in the miniscope experiments). Nevertheless, a subset of ROIs were only active during vocalization and not during USV playback in all (4/4) mice, indicating that expressive and receptive phases of vocal communication can engage distinct pIns ROIs. Finally, we compared the activation times of these vocalization-modulated ROIs in the pIns with those of vocalization-modulated ROIs in the AuC from a previous study.^31^ This comparison revealed that the modulation prior to vocal onset occurred earlier in the pIns than in the AuC (Figure 4D, Wilcoxon, *p* < 0.001). In summary, the 2p imaging approach used here revealed that a greater proportion of pIns ROIs were active during USV production and playback than detected using miniscopes, presumably reflecting the enhanced sensitivity of 2p imaging methods. Nonetheless, 2p imaging confirmed that the subsets of ROIs activated during these two conditions were largely non-overlapping while also showing that female odorants modulate male pIns activity evoked by the auditory presentation of other males’ USVs.

### The pIns links the auditory thalamus with a vocal gating region in the PAG

**Figure 5.**
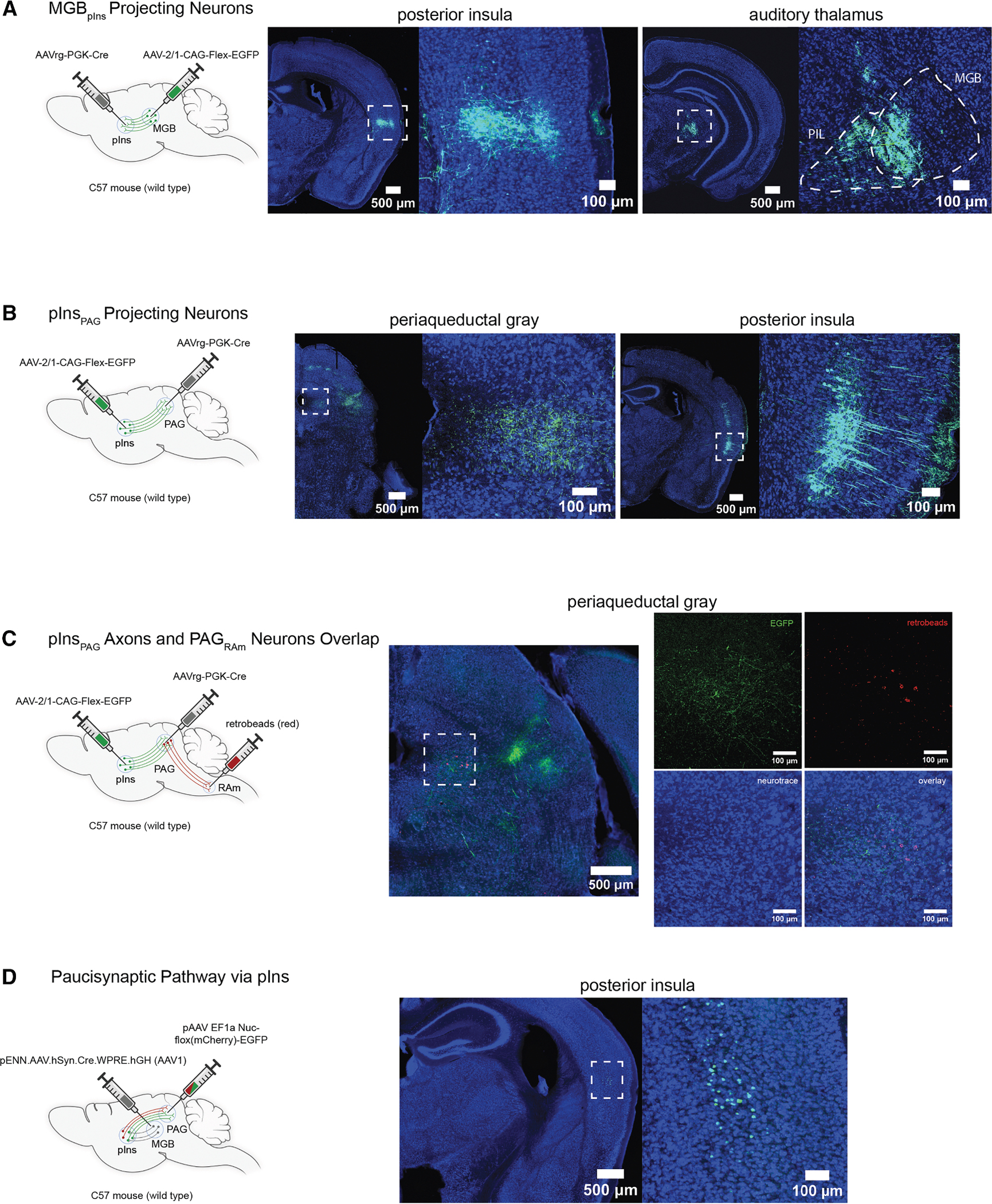
The posterior insula links the auditory thalamus with a vocal gating region in the PAG (A) Experimental design to label MGB_pIns_ projecting neurons (left); axon terminals in pIns (middle, left scale bar: 500 μm, right scale bar: 100 μm); cell bodies in MGB/PIL region (right, left scale bar: 500 μm, right scale bar: 100 m). (B) Experimental design to label pInsPAG projecting neurons (left); axon terminals in PAG (middle, left scale bar: 500 μm, right scale bar: 100 μm); layer 5 cell bodies in pIns (right, left scale bar: 500 μm, right scale bar: 100 μm). (C) Experimental design to identify projections to vocal gating region in the PAG (left); axon terminals of pIns_PAG_ projections (green) and cell bodies of PAG_RAm_ neurons (red) (left scale bar: 500 μm, right scale bar: 100 μm). (D) Experimental design to identify pInsPAG neurons that receive direct inputs from MGPpIns neurons (left); layer 5 pInsPAG neurons that expressed a colorflipper virus and switched from red to green due to the presence of Cre (right) (left scale bar: 500 μm, right scale bar: 100 μm). pIns, posterior insula; MGB, auditory thalamus; PIL, posterior intrathalamic nucleus; PAG, periaqueductal gray; RAm, nucleus retroambiguus.

Previous neuronal tracing studies with fluorescent markers provide evidence that neurons in the auditory thalamus (medial geniculate body [MGB]) make axonal projections to the pIns.^13–15^ To further characterize this projection, we injected retrograde AAV-Cre (AAVrg-Cre) in the pIns and AAV-flex-GFP in the MGB (Figure 5A, left, *N* = 3). This approach resulted in robust GFP expression in cell bodies in the medioventral part of the MGB (MGB_pIns_) and in the posterior intralaminar thalamic nucleus (posterior intrathalamic nucleus [PIL]), which is adjacent to the MGB and is implicated in social, maternal, and sexual behaviors^34–37^ (Figure 5A, right). This intersectional approach also resulted in dense GFP labeling in axon terminals in layers 4–6 and sparse GFP labeling in layers 1–3 of the pIns (Figures 5A, middle, and S7A), confirming that thalamic regions, including the MGB and PIL, are a source of input to the pIns.

Prior studies have established that neurons in the caudolateral PAG (clPAG) gate USV production in male mice through their axonal projections to vocal premotor neurons in the nucleus retroambiguus (PAG_RAm_).^24,38^ We explored whether the pIns innervates this vocal gating region of the clPAG by injecting AAV retro-Cre in the PAG and AAV-FLEXed GFP in the pIns (Figure 5B, left, *N* = 4). This intersectional approach resulted in robust GFP expression in cell bodies in the pIns, especially in layer V (Figure 5B, middle and right). We extended this approach by combining this intersectional approach with injections of retrogradely transported fluorescent latex microbeads into the nucleus RAm (Figure 5C, left). This approach revealed that the axon terminals of PAG-projecting pIns neurons overlapped with the region of the clPAG that projects to the RAm (Figure 5C, right, *N* = 2). Finally, we tested whether the region of the pIns that projects to the PAG_RAm_ also receives input from the auditory thalamus (Figure 5D, left, *N* = 3). We injected AAV1-Cre into the MGB, resulting in Cre expression in pIns neurons postsynaptic to the MGB, and injected a retrogradely transported AAV into the clPAG, resulting in expression in the pIns of a fluorescent reporter that flips from red to green in a Cre-dependent manner (AAVrg-colorflipper). This approach resulted in GFP expression in cell bodies of layer 5 neurons in the pIns (Figure 5D, right). These results indicate that the pIns directly links the auditory thalamus with the vocal gating region in PAG.

To probe a possible causal role in USV production, we conducted a new set of chemogenetic experiments in which we bilaterally injected male mice with a control virus (AAV2/9-hsyn-mCherry), inhibitory DREADDs (AAV2/9-hsyn-hM4Di-mCherry), or excitatory DREADDs (AAV2/9-hsyn-hM3Dq-mCherry) targeting the pIns. Mice were then administered either saline or clozapine N-oxide (CNO) on separate days and exposed to a female for 10 min while we monitored their vocal behavior. Across 22 male mice, 8 were excluded for not vocalizing during the test sessions (1 control, 4 inhibitory, and 3 excitatory males). Our results did not show significant changes in the rate of USV syllable production across conditions (Figure S6C). While these results suggest that pIns activity is not necessary for normal levels of USV production, they are also consistent with prior studies that show that frontal cortical regions play, at best, a very limited role in USV production in male mice.^39,40^

### The pIns communicates with other brain regions for social behavior

**Figure 6.**
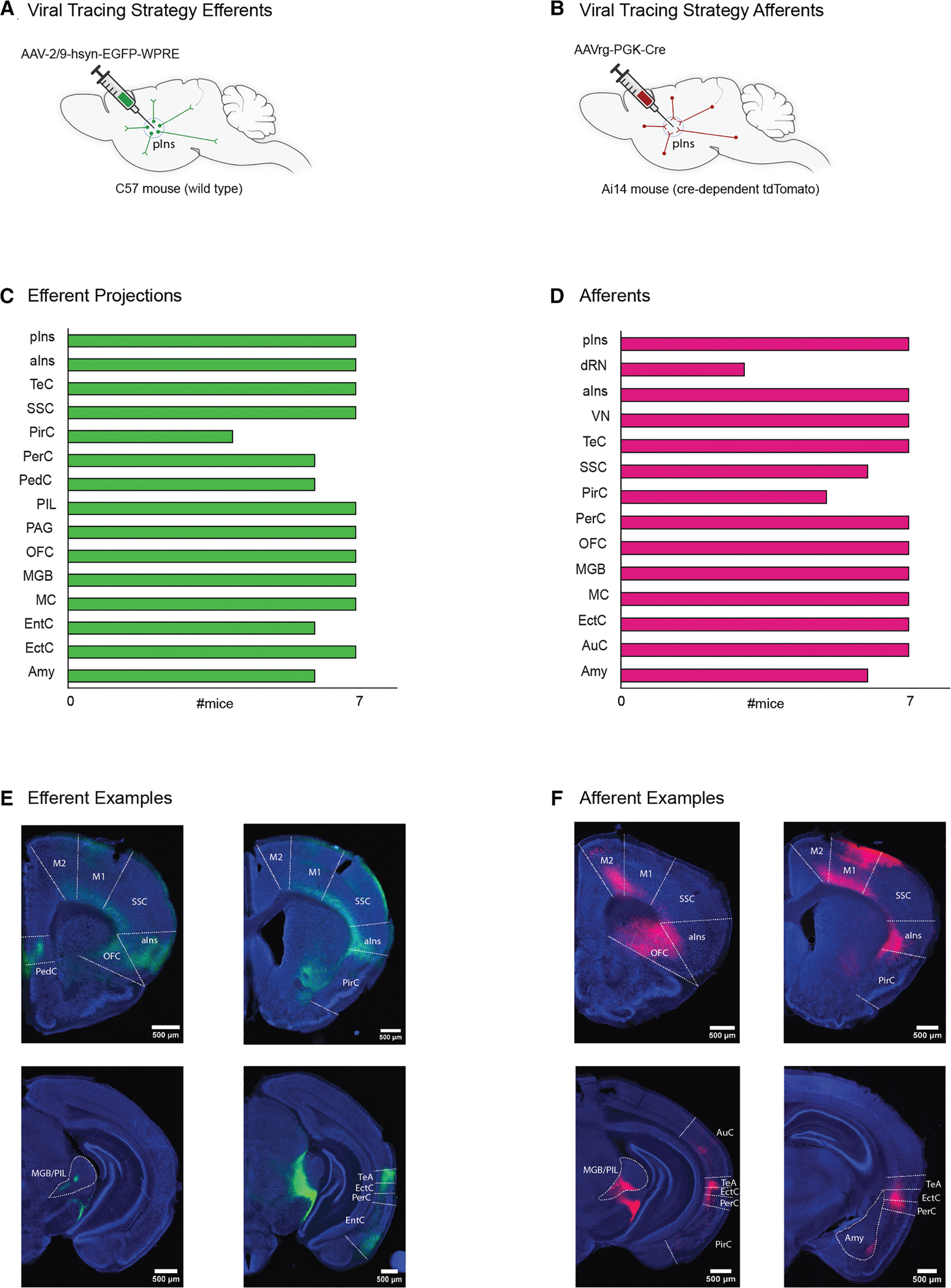
The posterior insula communicates with other brain regions for social behavior (A and B) Experimental approach to trace efferents (green) and afferents (red) of the pIns. (C) Identified efferents in seven mice. (D) Identified afferents in seven mice. (E) Efferent examples. Scale bar: 500 μm. (F) Afferent examples. Scale bar: 500 μm. pIns, posterior insula; aIns, anterior insula; TeC, temporal association cortex; PirC, piriform cortex; PerC, perirhinal cortex; PIL, posterior intrathalamic nucleus; PAG, periaqueductal gray; OFC, orbitofrontal cortex; MGB, medial geniculate body; M1/M2, primary and secondary motor cortex; EntC, entorhinal cortex; EctC, ectorhinal cortex; Amy, amygdala; dRN, dorsal raphe nucleus; VMN, ventromedial thalamic nucleus; SSC, somatosensory cortex; AuC, auditory cortex; PedC, peduncular cortex.

We also performed further viral tracing experiments to map the efferents and afferents of the pIns. In one set of mice (*N* = 7 mice, 4 male and 3 female), we injected AAV9 into the pIns of wild-type mice (*N* = 7) to express EGFP in pIns axons and putative axon terminals, which were distinguishable by varicosities (Figures 6A and S7B–S7D). In another set of mice (*N* = 7 mice, 4 male and 3 female), we injected AAVrg-Cre into the pIns of transgenic Ai14 mice (*N* = 7) to express tdTomato in neurons afferent to the pIns (Figure 6B). These viral tracing experiments revealed reciprocal connections between the pIns and other cortical regions, including the anterior insula, amygdala, motor cortex (primary motor cortex [M1] and secondary motor cortex [M2]), orbitofrontal cortex, piriform cortex, and rhinal cortex (Figures 6C–6F). Additionally, the pIns made reciprocal connections with the temporal association cortex, a region involved in encoding ultrasonic pup vocalizations,^41^ and with the MGB/PIL. This approach also revealed that the pIns made a variety of non-reciprocal connections, especially with subcortical regions. These include efferents from the pIns to the PAG and afferents from the dorsal raphe nucleus and the ventromedial thalamic nucleus to the pIns (Figures 6C and 6E, bottom left). The current mapping results are consistent with an earlier study indicating that the pIns is bidirectionally connected with many cortical regions and mostly unidirectionally connected with subcortical connections.^42^

## DISCUSSION

Here, we used calcium imaging in freely courting male and female mice to characterize the activity of pIns neurons during vocal communication. In male mice, we identified two mostly distinct but spatially intermingled neuronal populations in the pIns that increased their activity during vocal communication. One population increased its activity prior to and during USV production in both hearing and deaf mice, consistent with a premotor or proprioceptive signal. Another population, which responded to USVs produced by nearby male mice engaged in courtship, was detected only in hearing and not deaf mice; similar responses were also detected in the pIns of female mice interacting with a vocalizing male. Notably, this USV-responsive population was only weakly excited by USV playback when mice were placed in social isolation, indicating that USV responsiveness in the pIns is augmented by non-auditory social cues. In fact, two-photon calcium imaging in head-fixed male mice revealed that female odorants could enhance USV responsiveness. A combination of intersectional and conventional tracing methods indicated that the pIns, and specifically layer 5 neurons, bridges the auditory thalamus with a region of the PAG that gates USV production. In summary, the pIns integrates auditory, olfactory, and vocalization-related signals to encode expressive and receptive aspects of vocal communication in a manner that is sensitive to social context.

While the pIns is well known as a site for the auditory encoding of conspecific vocalizations,^12^ pointing to a receptive function, here we found that the pIns is remarkably active during USV production, consistent with an expressive function. Specifically, activity in the pIns increased prior to the onset of USV production and remained elevated throughout the vocal bout. Moreover, similar patterns of vocalization-related activity were observed in hearing and congenitally deaf mice. The pre-vocal, hearing-independent nature of this vocalization-related activity is consistent with a motor-related signal. Indeed, a major afferent to the pIns identified here and in earlier studies^42^ is the M2, a region that displays vocalization-related activity^43,44^ and is a source of motor-related corollary discharge signals to the primary AuC.^29,30^ Another possibility is that the pIns integrates proprioceptive signals originating from respiratory and vocal muscles, although neither the somatosensory cortex nor the thalamus was strongly labeled by the intersectional, retrograde tracing methods we employed. Because courtship USVs of male mice are typically produced in response to female odorants,^20^ the pre-vocal activity in the pIns could also be linked to olfactory signals, which could be transmitted to the pIns from the piriform cortex and amygdala. However, delivering female odorants to a head-fixed male did not modulate pIns activity in the absence of subsequent USV production. A remaining possibility is that the pre-vocal signature in the pIns reflects signals related to the decision to vocalize, which could be transmitted to the pIns from the orbitofrontal cortex^45^ or from the anterior insula, the latter of which receives input from the medial prefrontal cortex,^46^ a region directly linked to vocal production in rats, mice, and monkeys.^40,47,48^ In summary, a distinct subset of pIns neurons are activated during vocal expression, mostly likely reflecting signals linked to vocal motor production or the decision to initiate and sustain USV production.

Previous studies found that the pIns responded to pure tones in mice^13,14^ and to vocalizations of conspecifics in rhesus monkeys.^12^ While these studies point to the pIns as a site where vocalizations could be encoded in a socially salient manner, they monitored neural activity of playback stimuli delivered to head-fixed subjects in social isolation. An important advance of the current study is the analysis of pIns neurons during more naturalistic vocal communication involving several mice. Courtship USVs of male mice are typically produced in response to female odorants and render females more receptive to mating.^20,23,24^ The male’s USVs—given their pro-mating effects—can be regarded as adaptive signals that convey information about the male’s presence and reproductive fitness to an intended female target. However, as with other vocalizations, the courting male’s USVs convey information to any nearby animals that can hear them, including rival males. Here, we created a social-vocal context in which a courting male’s USVs could be monitored by both a female mouse, the male’s intended courtship target, and a nearby eavesdropping male. This approach revealed that hearing the courting male’s USVs increased activity in neurons in the pIns in both the female and the eavesdropping male. This USV-evoked increase in activity was not simply a consequence of auditory stimulation, as USV playback evoked much less activity in the pIns when the female or the eavesdropping male was placed in social isolation. Instead, our results indicate that the pIns encodes male courtship USVs in a socially salient manner, consistent with prior studies that implicate the insula more generally in salience detection.^5,49,50^

The current study identifies female odorants as an important social cue that augments pIns activity in a male listening to another male’s USVs. Specifically, multiphoton imaging in head-fixed male mice revealed that exposure to female odorants augments responses in a male’s pIns to USV playback. Consistent with prior anatomical studies,^6,42,51^ tracing experiments conducted here show that the pIns receives direct inputs from the piriform cortex and amygdala, providing a pathway by which odorants could modulate USV responses. More broadly, odorants from mouse pups can modulate auditory cortical responses in dams to pup cries,^52–54^ and thus, odorant-dependent modulation may reflect a more general feature of the cortical representation of vocal sounds in the mouse cortex. Two-photon imaging in the pIns of socially isolated mice also revealed that a larger subset of neurons were modulated by USV playback than when the same region was imaged with 1p miniscopes in socially isolated, unrestrained conditions. This could reflect the higher sensitivity of 2p methods or a heightened state of arousal in the head-fixed male that increases responses to auditory stimuli. Nevertheless, our results indicate that female odorants enhance the responsiveness of pIns neurons in male mice listening to the courtship USVs of other males.

The present study also underscores the pivotal position of the pIns in the vocal sensorimotor hierarchy. The pIns is partly defined as a region that receives input from the auditory thalamus.^15^ Our results extend these findings by elucidating that layer 5 neurons in the pIns receive direct input from the auditory thalamus and make axonal projections to the USV-gating region in the PAG.^24,38^ Whether these axons project directly to USV-gating neurons is unknown, but prior intersectional tracing studies from our lab suggest that they predominantly target local interneurons that provide inhibitory input onto USV-gating neurons in the PAG.^24^ In this framework, activity evoked in the pIns by listening to another male’s USVs could serve to suppress USV production in the listener. However, a purely suppressive effect of the pIns on vocal gating neurons in the PAG cannot account for how activity in some pIns neurons increases before and during USV production. Therefore, an important goal of future studies will be to establish the identity, connectivity, and function of pIns neurons that are active during expressive and receptive phases of social-vocal communication.

Effective social communication depends on establishing a correspondence between expressive and receptive aspects of communication signals. The current study shows that the pIns is a site where both expressive and receptive aspects of vocal signals are encoded, albeit in largely distinct neuronal populations. These observations confirm and extend a recent study in humans^16^ showing that the pIns is active during speech production and perception. Similar to primary auditory cortical neurons, we found that a population of pIns neurons that were responsive during vocal reception was suppressed during vocal production.^16,31,55–59^ However, unlike the primary AuC, an even larger subset of pIns neurons were strongly excited during vocal production, and this excitation arose earlier relative to vocal onset.^31^ Therefore, the insula contains both expressive and receptive representations of vocal sounds, which could help to establish a sensorimotor correspondence that facilitates communication.

### Limitations of the study

While this study provides novel insights into how the pIns encodes socially relevant vocal behaviors, several limitations should be considered. First, our findings are correlative in nature and did not demonstrate a causal role for the pIns in vocal communication. Second, while we identified distinct populations of ROIs associated with vocal expression and reception, we did not perform systematic cell-type-specific labeling or manipulation, leaving open questions about the molecular identity and functional roles of these subpopulations. Additionally, although tracing studies revealed a clear anatomical pathway linking the auditory thalamus, pIns, and PAG, the precise functional impact of these connections remains to be determined.

While our chemogenetic manipulation experiments in the pIns of vocalizing males support the conclusion that pIns activity is not necessary for normal levels of USV production, prior studies have established that the male’s “decision” to emit vocalizations depends on a complex and hierarchical interplay between subcortical regions converging on USV-gating neurons in the midbrain PAG.^24,38,60^ Therefore, this negative result does not exclude the possibility that the pIns makes a contribution to this overall decision, perhaps by influencing more subtle aspects of precisely when USVs are emitted during a social encounter. Ultimately, a further range of gain- and loss-of-function manipulations will be needed to determine whether the pIns influences expressive or receptive phases of USV communication.

Future studies using cell-type-specific manipulations and circuit-level interrogation will be essential to establish causal roles and further unravel how the pIns integrates social, auditory, and motor information during vocal communication.

## RESOURCE AVAILABILITY

### Lead contact

Requests for further information and resources should be directed to and will be fulfilled by the lead contact, Thomas Pomberger (thomas.pomberger@duke.edu).

### Materials availability

This study did not generate new unique reagents.

### Data and code availability

All data reported in this paper will be shared by the lead contact upon request. This paper does not report original code. Any additional information required to reanalyze the data reported in this paper is available from the lead contact upon request.

## STAR★METHODS

### EXPERIMENTAL MODEL AND STUDY PARTICIPANT DETAILS

#### Animals statement

All experiments were conducted according to a protocol approved by the Duke University Institutional Animal Care and Use Committee (protocol # A183–23-09 (1)).

#### Animals

For calcium imaging (1-photon and 2-photon) and neuronal tracing experiments, the following mouse lines from Jackson labs were used: C57 (C57BL/6J, Jackson Labs, 000664, 40 males, 14 females), Tmc1(Δ) (courtesy of Jeffery Holt, Harvard University, 5 males) and Ai14 (B6.Cg-Gt(ROSA)26Sortim14(Cag-tdTomato)Hze/J, Jackson Labs, 007914, 4 males, 3 females). Mice were housed in 12/12 h day/night cycle and they had permanent access to food (pellets) and water. All mice used for these experiments were between 90 and 180 days old and were housed with other conspecifics of the same sex. Mice were allocated to experimental groups based on genotype and experimental need. Group sizes were determined based on prior published studies using similar imaging and tracing methods, with a minimum of 2–5 animals per condition to ensure reproducibility. No randomization or blinding was applied during group assignment. We examined the potential influence of sex on experimental outcomes in the neuronal tracing experiments and in experiments assessing vocal receptivity. No significant differences between males and females were found. However, only male mice were used in vocal expression experiments, as female USVs are typically produced in different social contexts than courtship and are not as robust or reliable as male vocalizations in this setting.

### METHOD DETAILS

#### Lens implantation and baseplating

One surface of a GRIN lense (4mm length, 1mm diameter, Inscopix) was covered with a silk-fibroin-virus mixture (1 part virus, 1 part silk fibroin) either the day before surgery and kept overnight at 4°C or 30 min before implantation as described in Jackman et al. 2018.^64^ Mice were then anesthetized (1.5%–2% isoflurane), and the pIns was targeted for injection. GRIN lenses were then implanted 0.1mm above pIns target location and were fixed to the skull using Metabond (Parkell) and dental cement (Ortho-Jet). We covered the lens with body-double and an additional layer of dental cement to protect it from damage. After a recovery period of 4–6 weeks, a baseplate was cemented on top of the animal and imaging experiments were conducted starting 3–7 days after baseplating.

#### USV recording and analysis

USVs were recorded using ultrasonic microphones (Avisoft, CMPA/CM16) amplified (Presonus TubePreV2), and digitized at 192 kHz/250 kHz (RZ6 Multi I/O Processor from Tucker Davis and a Power1401 CED board, Spike2) during 1p- and 2p-imaging, respectively. USVs were detected using Mupet.^65^ USV bouts were defined by a minimum duration of 500ms and a minimum interbout duration of 2 s. Custom MATLAB code was used to visualize each detected bout, and on- and offsets were manually adjusted if necessary.

#### Playback stimulus presentation

We used pre-recorded USVs from freely interacting males and females. Ultrasonic loud speakers (ES1 SN: 4907, Tucker Davis Technologies) were used to present these stimuli. Four different USV bouts with a length of 2–8 s were presented during the 1p-imaging experiments (10 presentations per stimulus, pseudorandomized order, 40 presentations in total). Six different USV bouts with a length of 2 s were presented during the 2p-imaging experiments (20 presentations per stimulus, pseudorandomized order, 120 presentations in total).

#### Behavior recording and analysis

All experiments were conducted under infrared light (IR Illuminator, model: YY-IR30W, LineMax). We used a webcam (HD 1080p, Logitech) from which we removed the infrared filter to monitor the behaviors of the mice. Animal pose estimations were acquired by using Deeplabcut.^66^ We then used custom MATLAB code to calculate speed and acceleration of an animal. Runnning bouts were defined as follows: minimum duration 0.5sec, interbout duration 1sec. Acceleration bouts were defined as follows: minimum duration 0.25sec. Area dimensions of the arena were acquired manually and we used video frames to convert pixels into metric values.

#### One-photon imaging

On the day of testing, a miniature miscroscope (UCLA miniscope V4) was mounted on the baseplate of the animal and fixated in place by a screw before the animal was placed into one of the two chambers of the two-chamber assay. Calcium data was acquired using the provided open-source software for UCLA miniscopes V4 which synchronized its recording times by sending out a TTL pulse to the audio recording system each time a frame was acquired. After an acclimation period of 3–5 min, the animal was exposed to other conspecifics and playback stimuli. Video, audio and calcium signals were recorded as the mouse freely interacted with the presented stimuli. We extracted ROIs from the miniscope videos using minian,^28^ an automated pipeline that performs motion correction and ROI extraction via CNMF-E, and allows for visual inspection of ROIs afterward. The resulting calcium signal was analyzed with custom MATLAB codes.

#### Two-photon imaging

Prior to 2-photon calcium imaging we implanted titanium Y-headbars on mice using Metabond (Parkell) after they underwent surgery for GRIN lens implantation as described above. Mice were head-fixed on a radial treadmill and habituated for at least one week before the experiment was conducted. The baseplate was filled with carbomer gel (refractive index 1.4) and signal were recorded by a 10x/0.45NA water immersion objective (Nikon). We used a titanium sapphire laser (MaiTai DeepSee, 920nm, Neurolabware) with a laser power of 100mW. Recordings were performed in darkness. Data was acquired using Scanbox (sampling rate 15.49 Hz; 512 × 512 pixels) that sent out a TTL pulse to Spike7 audio-recording system each time a frame was acquired. Suite2p^61^ was used to extract individual calcium signals and subsequent data analysis was performed by custom MATLAB code. Extracted ROIs were manually inspected.

#### Viruses and tracers

We used the following viruses and tracers: AAV2/9-*syn*-jGCaMP8s-WPRE (Addgene), AAVrg-PGK-Cre (Addgene), AAV-2/1/CAG-Flex-EGFP (Addgene), pENN.AAV.hsyn.Cre.WPRE.hGH (AAV1, Addgene), pOOTC1032 – pAAV-EF1a-Nuc-flox(mCherry)-EGFP, AAV9-hSyn-mCherry (Addgene), AAV9-hSyn-hM3D(Gq)-mCherry (Addgene), AAV9-hSyn-hM4D(Gi)-mCherry (Addgene), and Red Retrobeads IX (LumaFluor). We injected into the following coordinates relative to bregma: pIns, AP = −1.05mm, ML = 3.80mm, DV = −3.50mm; MGB, AP = −2.90mm, ML = −1.75mm, DV = −3.40mm; PAG = −4.7mm, ML = 0.70mm, DV = −1.75mm; RAm: AP = −8.05mm, ML = 1.00mm, DV = −5.20mm. Coordinates were achieved via a digital stereotaxic instrument (RWD) and viruses were pressure-injected with a Nanoject III (Drummond) at a rate of 1nL/s.

#### Post-hoc visualization of viral labeling

Mice were deeply anesthetized with isoflurance and then transcardially perfused with ice-cold 4% paraformaldehyde in 0.1 M phosphate buffer, pH 7.4 (4% PFA). Dissected brain samples were postfixed overnight in 4% PFA at 4°C, cryoprotected in a 30% sucrose solution in PBS at 4°C for 48 h, frozen in Tissue-Tek O.C.T. Compound (Sakura), and stored at −80°C until sectioning. Brains were cut into 100 μm coronal sections, rinsed 3x in PBS, and processed for 24 h at 4° with NeuroTrace (1:500, Invitrogen) in PBS. To increase fluorescence of jGCaMP8s in brain slices we added primary antibody (Chk pAb to GFP, ab13970, Abcam) to NeuroTraces, rinsed the samples 3x in PBS and processed with secondary antibody (Anti-Chicken IgY, 488, 703–545-155, Jackson ImmunoResearch). Tissue sections were rinsed again 3x in PBS, mounted on slides, and coverslipped with Fluoromount-G (Southern Biotech). After drying, slides were imaged with a 10× objective in a Zeiss 700 laser scanning confocal microscope and a Keyence microscope (BZ-X810, All-in-One Fluorescence Microscope).

#### Chemogenetic manipulations

Animals were injected with the corresponding DREADDs viruses or controls (see Viruses and Tracers) bilaterally into the pIns using 150 μL per injection. After two weeks, animals were injected intraperitoneally with saline or CNO 20 min before exposure to a female. Courtship behavior and the resulting vocalizations were recorded for 10 min. Animals were treated with saline for 2 days followed by a 3^rd^ day were CNO was injected.

### QUANTIFICATION AND STATISTICAL ANALYSIS

The number of animals used in each condition is provided in the figure legends (e.g., *N* = 5 hearing males, *N* = 5 deaf males and *N* = 5 females for miniscope imaging; *N* = 4 males for 2-photon imaging), and n refers to the number of animals unless otherwise noted. The percentage and spatial distribution of responsive ROIs, as well as their classification (e.g., ROIs_Voc_, ROIs_EDrop_), are detailed in the results, main Figures (e.g., Figures 1, 2, 3, and 4), and Supplemental Figures (e.g., S2–S5). Principal component analysis (PCA) and cosine similarity analyses were used to examine population vector structure and separability across behavioral states. All analyses were performed using automated and unbiased pipelines in custom MATLAB code. Assumptions for non-parametric tests were considered met, and no data were excluded unless specified. Formal power analyses were not conducted. ROIs were manually inspected for quality following automated extraction using minian^28^ (for 1-photon) or suite2p^63^ (for 2-photon). Spikes from the extracted ROIs were inferred based on Friedrich et al. 2017.^67^

#### Detection of responsive ROIs

All ΔF/F calcium traces were z-scored prior to each analysis and were presented in units of standard deviation. We quantified responses of each ROI during miniscope recordings using a receiver operating characteristic (ROC) analysis, which has been applied previously to detect responses during natural behavior.^68–70^ ROC curves for each ROI were calculated by comparing distributions of calcium responses across all vocal bouts and during baseline (defined as the mean before and after vocal onset), using a moving criterion defined as (max – min)/100 across both distributions. The area under the ROC curve (AUC) was then compared to a null distribution generated from 1000 shuffled iterations. ROIs with AUC values below the 2.5th percentile or above the 97.5th percentile were classified as significantly suppressed or excited, respectively. Playback responses during 1-photon and 2-photon calcium imaging, as well as changes in running speed or acceleration, were analyzed using two-sided Wilcoxon rank-sum or signed-rank tests as appropriate. Individual average calcium traces were baseline-subtracted prior to visualization.

#### Temporal modulation analysis

To characterize the temporal structure of population responses in the posterior insula during vocal behavior, we applied a temporal modulation analysis framework adapted from methods described in Hernández et al. 2010^71^ and Rossi-Pool et al. 2017.^72^ Specifically, we quantified temporal variability in neuronal population responses by computing, in overlapping time windows, the percentage of ROIs showing significant excitation or suppression relative to baseline activity. The analysis slides a fixed-size time bin across the trial using a defined step and calculates the mean *Z* score for each ROI within each bin. ROIs are classified as significantly excited or suppressed if their mean *Z* score exceeds +0.5 or falls below −0.2, respectively. The percentage of excited and suppressed ROIs is calculated for each bin, producing a time series that captures the dynamic modulation of the population.

#### Population vector and cosine similarity analysis

To characterize the structure of neural population activity in the posterior insula during vocal communication, we constructed population vectors from simultaneously recorded ROI activity during each behavioral session as described in Carillo-Reid et al. 2019.^73^ Each vector corresponded to the coactivity state of all ROIs at a given time point (frame) and was represented as a binary array. We computed the similarity between all population vectors within and across conditions using the cosine similarity metric, defined as the normalized inner product between pairs of vectors. Similarity matrices were generated by computing pairwise cosine similarities between vectors and were sorted by behavioral condition to visualize structured coactivity patterns. Principal component analysis (PCA) was applied to the population vectors to reduce dimensionality and visualize clustering of ensemble activity in state space. To quantify condition-specific population structure, we computed the mean cosine similarity between all population vectors within each behavioral context and compared these to the similarity across contexts using a non-parametric Kruskal-Walis test. All analyses were implemented in custom MATLAB scripts and visualized as similarity heatmaps, PCA projections, and boxplots (e.g., Figures 1I–1K; S5).

#### LSTM-based behavioral state decoding

For each recording session we performed a principal component analysis on all ROIs and used the first 24 principle components (PCs) as the input layer to our model. We then divided each recording session into two datasets: The training dataset contained 85% of vocal bouts while the test dataset contained 15% of vocal bouts. We then created a long short-term memory network using MATLAB that we trained on the training dataset to decode vocal bouts by PC activity. Next, we applied the decoder to the original test data and a control dataset where we randomly shuffled the USV bout appearances. The resulting decoding accuracies for each session were quantified by a standard two-sided Wilcoxon rank-sum test.

## Supplementary Material

1

SUPPLEMENTAL INFORMATION

Supplemental information can be found online at https://doi.org/10.1016/j.celrep.2025.115850.

## Figures and Tables

**KEY RESOURCES TABLE:** 

REAGENT or RESOURCE	SOURCE	IDENTIFIER

Antibodies		

Chk pAb to GFP	Abcam	Cat#ab13970; RRID: AB_300798
Anti-Chicken IgY, 488	Jackson ImmunoResearch	Cat#703-545-155: RRID: AB_2340375
Chk pAb to GFP	Abcam	Cat#ab13970; RRID: AB_300798
Anti-Chicken IgY, 488	Jackson ImmunoResearch	Cat#ab703-545-155; RRID: AB_2340375

Bacterial and virus strains		

AAV2/9-*syn*-jGCaMP8s-WPRE	Zhang et al. 2020^61^	Cat#162374 Addgene
AAVrg-PGK-Cre	Patrick Aebischer	Cat#24593-AAVrg Addgene
AAV-2/1-CAG-Flex-EGFP	Ian Wickersham	Cat#59331-AAV1 Addgene
pENN.AAV.hsyn.Cre.WPRE.hGH	James M. Wilson	Cat#105553-AAV1 Addgene
pOOTC1032-pAAV-EF1a-Nuc-flox(mCherry)-EGFP	Back et al. 2019^62^	Cat#112677-AAVrg Addgene
AAV2/9-*syn*-jGCaMP8s-WPRE	Zhang et al. 2020^61^	Cat#162374 Addgene
AAVrg-PGK-Cre	Patrick Aebischer	Cat#24593-AAVrg Addgene
AAV-2/1-CAG-Flex-EGFP	Ian Wickersham	Cat#59331-AAV1 Addgene
pENN.AAV.hsyn.Cre.WPRE.hGH	James M. Wilson	Cat#105553-AAV1 Addgene
pOOTC1032-pAAV-EF1a-Nuc-flox(mCherry)-EGFP	Back et al. 2019^62^	Cat#112677-AAVrg Addgene
pAAV-hSyn-mCherry	Karl Deisseroth	Cat#114472-AAV9 Addgene
pAAV-hSyn-hM3D(Gq)-mCherry	Bryan Roth	Cat#50474-AAV9 Addgene
pAAV-hSyn-hM4D(Gi)-mCherry	Bryan Roth	Cat#50475-AAV9 Addgene

Chemicals, peptides, and recombinant proteins		

Red Retrobeads^™^	LumaFluor	Cat#IX
Red Retrobeads^™^	LumaFluor	Cat#IX

Experimental models: Organisms/strains		

C57BL/6J	Jackson Labs	Cat#000664
Tmc1(Δ)	Jeffery Holt (Kawashima et al. 2011^32^)	N/A
B6.Cg-Gt(ROSA)26Sortim14(Cag-tdTomato)Hze/J (Ai14)	Jackson Labs	Cat#007914
C57BL/6J	Jackson Labs	Cat#000664
Tmc1(Δ)	Jeffery Holt (Kawashima et al. 2011^32^)	N/A
B6.Cg-Gt(ROSA)26Sortim14(Cag-tdTomato)Hze/J (Ai14)	Jackson Labs	Cat#007914

Software and algorithms		

Minian	Denise Cai (Dong et al. 2022^28^)	GitHub - denisecailab/minian: miniscope analysis pipeline with interactive visualizations
Suite2p	Kenneth D. Harris (Pachitariu et al. 2016^63^)	GitHub - MouseLand/suite2p: cell detection in calcium imaging recordings
MATLAB	Mathworks	N/A
Minian	Denise Cai (Dong et al. 2022^28^)	GitHub - denisecailab/minian: miniscope analysis pipeline with interactive visualizations
Suite2p	Kenneth D. Harris (Pachitariu et al. 2016^63^)	GitHub - MouseLand/suite2p: cell detection in calcium imaging recordings
MATLAB	Mathworks	N/A

## Highlights

- During courtship, USV production and reception activate largely distinct subsets of pIns ROIs
- Vocal production-related activity precedes sound onset and persists in congenitally deaf mice
- During courtship, receptive pIns responses require both vocal sounds and social context
- Layer 5 pIns neurons link auditory thalamus with midbrain vocal gating circuits
