# Supplementary material for: The mouse posterior insular cortex encodes expressive and receptive aspects of courtship vocalizations
Source: Cell Rep. Author manuscript; Available in PMC 2025 Jul 31. (PMC12312785; doi:10.1016/j.celrep.2025.115850)
Supplement: 1 [file NIHMS2092700-supplement-1.pdf]

Cell Reports, Volume *44*

Supplemental information

The mouse posterior insular cortex encodes expressive and receptive aspects of courtship vocalizations

Thomas Pomberger, Katherine S. Kaplan, Rene Carter, Autumn Wetsel, Thomas C. Harmon, and Richard Mooney

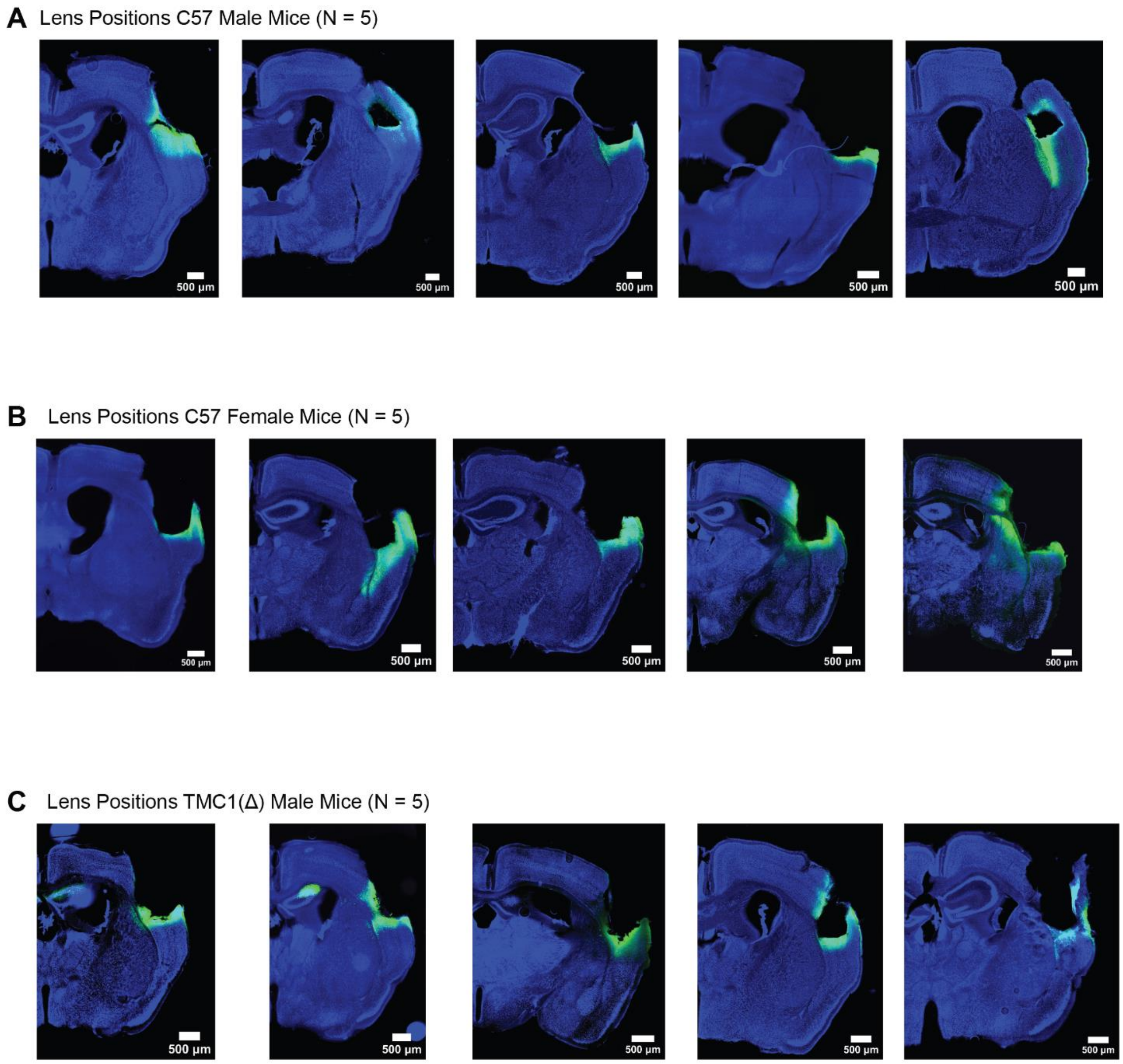

Supplemental Figure 1: Coronal sections of mouse brains (blue neurotrace staining) expressing GCaMP8s (green). (A) Lens positions of male C57 (hearing) mice; scale bar: 500 µm. (B) Lens positions of female C57 mice; scale bar: 500 µm. (C) Lens positions of male TMC1(Δ) (deaf) mice; scale bar: 500 µm.

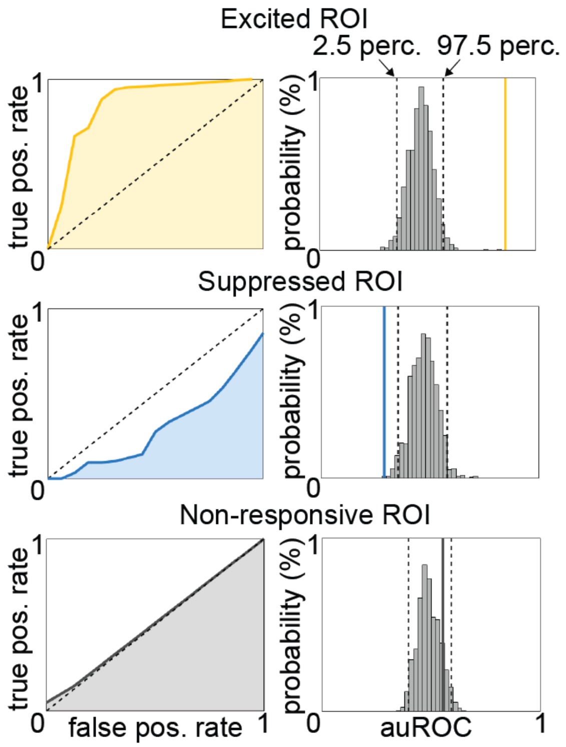

Supplemental Figure 2: Schematic examples of receiver-operator characteristic to quantify responsiveness of ROIs. Top, excited; middle, suppressed; bottom, non-responsive.

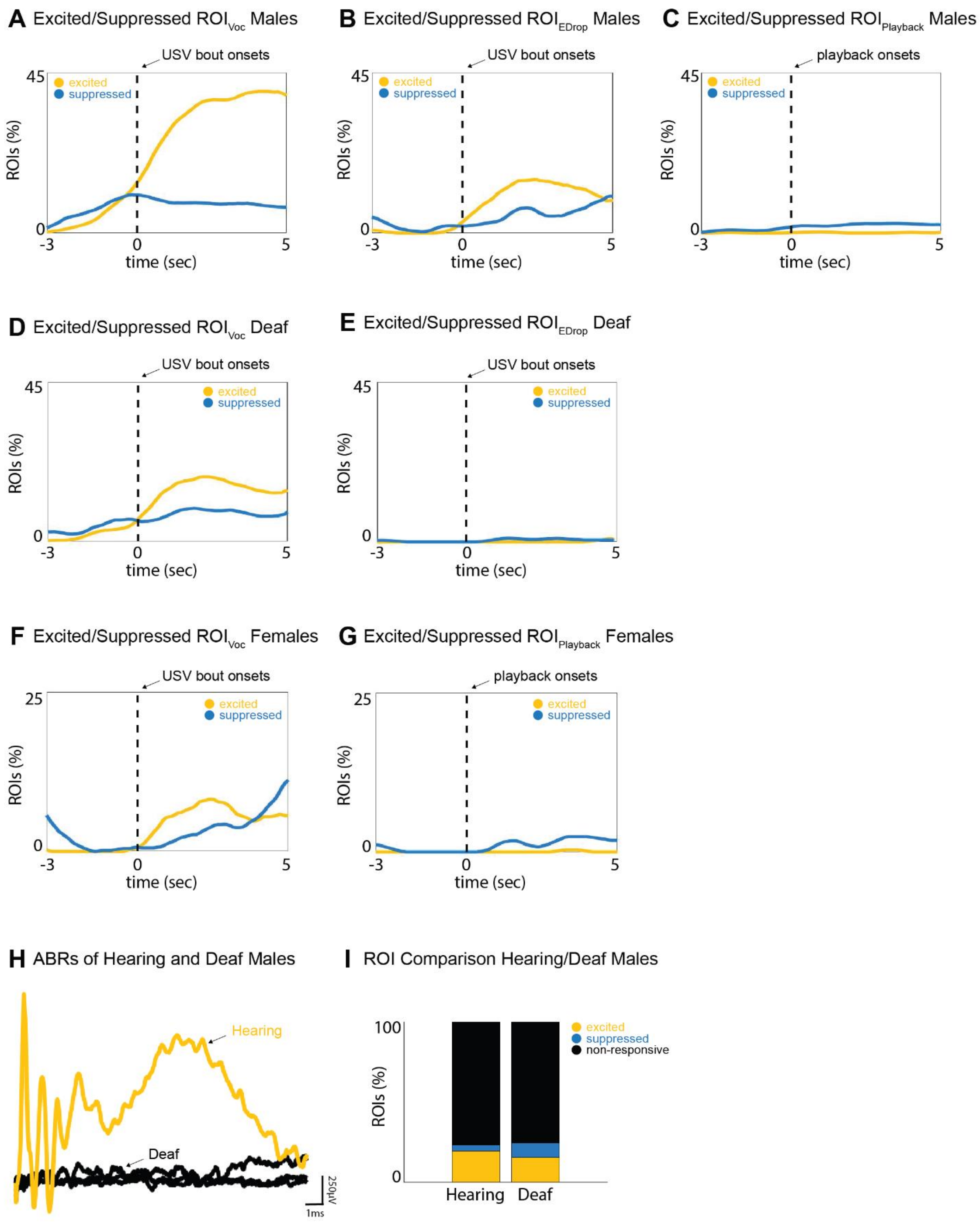

Supplemental figure 3: Percentage of active pIns ROIs over time, ABR responses and ROI comparisons. Vocalization (A), eavesdropping (B) and vocalization playback in hearing males (C). Percentage of active pIns ROIs over time during vocalization (D) and eavesdropping (E) in deaf males. Percentage of active pIns ROIs over time during male vocalization (F) and vocalization playback (G) in females. (H) Auditory brainstem responses in hearing (yellow) and deaf (black) males. (I) Percentage of excited, suppressed and non-responsive ROIs in hearing and deaf males during active vocalization.

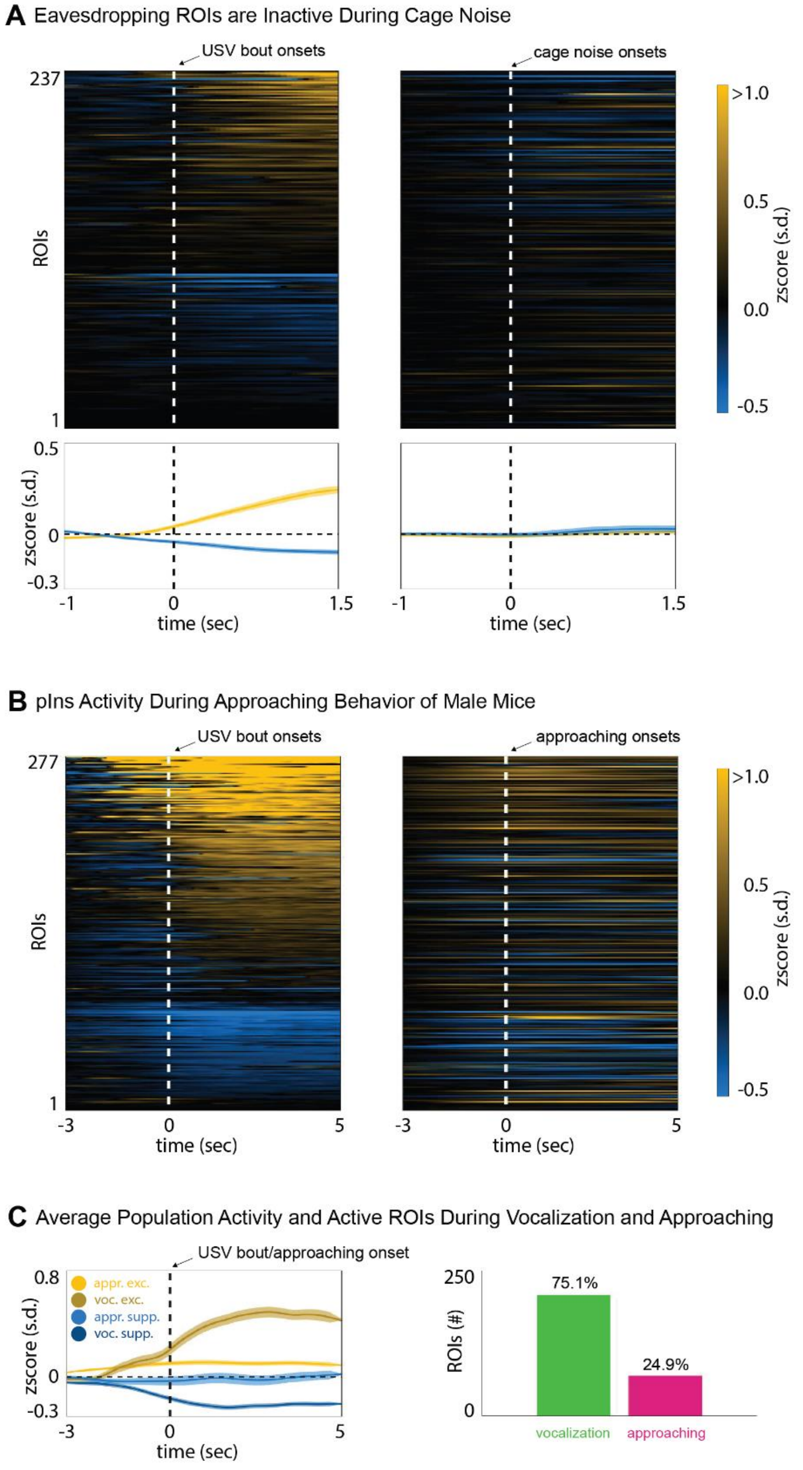

Supplemental figure 4: Social approach and cage noise do not account for the majority of ROI responses in pIns. (A) ROIs (z-scored for top panel) that are active during eavesdropping (left) are inactive during cage noise (right) (N = 6, 5 males, 1 female, mean ± SEM for bottom panels). (B) Comparison of

ROI activity during vocalization (left) and approaching towards a female (right) in male mice (N = 2 hearing & 3 deaf males, z-scored). (C) Overlap of overall population activities (mean ± SEM) from A (left) and relative amount of ROIs active during vocalization only (green) and during approaching (magenta).

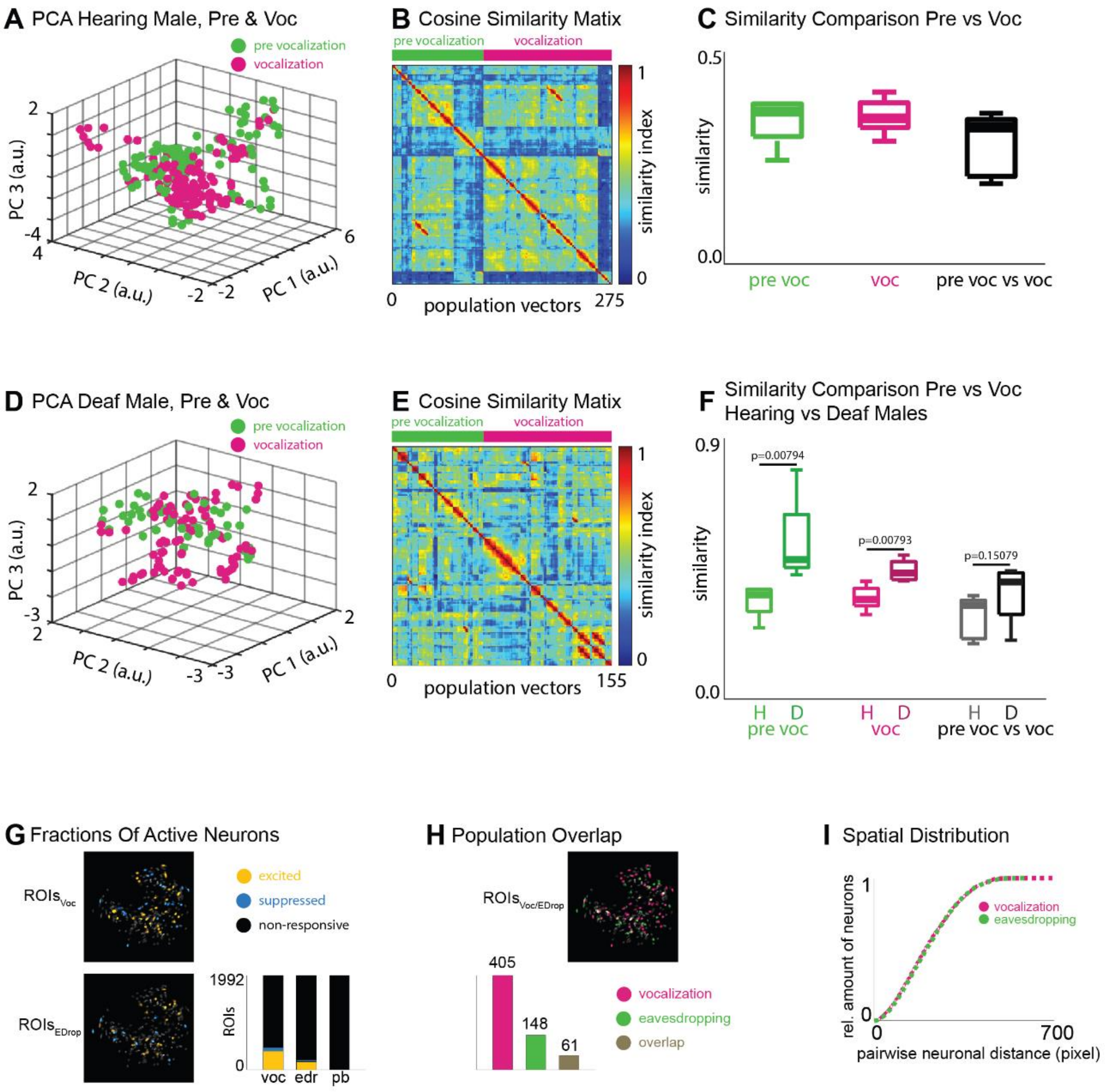

Supplemental figure 5: Population vector dynamics reveal strong similarities between hearing and deaf vocalizing males. (A) Principal component analysis (PCA) of population vectors from a representative hearing male that show co-active groups of ROIs during pre-vocalization (green) and vocalization (magenta) phases. (B) Sorted similarity map of the hearing male from (A) showing similarity between pre-vocalization and vocalization phases. (C) Cosine similarities of population vectors related to pre-vocalization and vocalization are similar for all mice (N = 5, Kruskal-Wallis, $p > 0.05$). (D) PCA of population vectors from a representative deaf male that show co-active groups of ROIs during pre-vocalization (green) and vocalization (magenta) phases. (E) Sorted similarity map of the deaf male from (D) showing similarity between pre-vocalization and vocalization phases. (F) Cosine similarities of population vectors related to pre-vocalization and vocalization are similar within groups of hearing and deaf males, but show higher similarity for deaf males (N = 10, 5 hearing & 5 deaf males, Wilcoxon). (H) Example field of views of $ROIs_{Voc}$ and $ROIs_{EDrop}$ (top and bottom left) and total amount of excited, suppressed and non-responsive ROIs in each context (voc = vocal expression, edr = eavesdropping, pb = playback). (G) Example field of view of

ROIs that are responsive to vocal expression or eavesdropping and their overlap (top). Total amount of responsive ROIs (N = 5 hearing males) in vocal expression (magenta), eavesdropping (green) or both contexts (brown). (J) Cumulative distribution function (Kolmogorov-Smirnoff, $p > 0.71$) of pairwise neuronal distances in pixel of $ROIs_{Voc}$ (magenta) and $ROIs_{EDrop}$ (green).

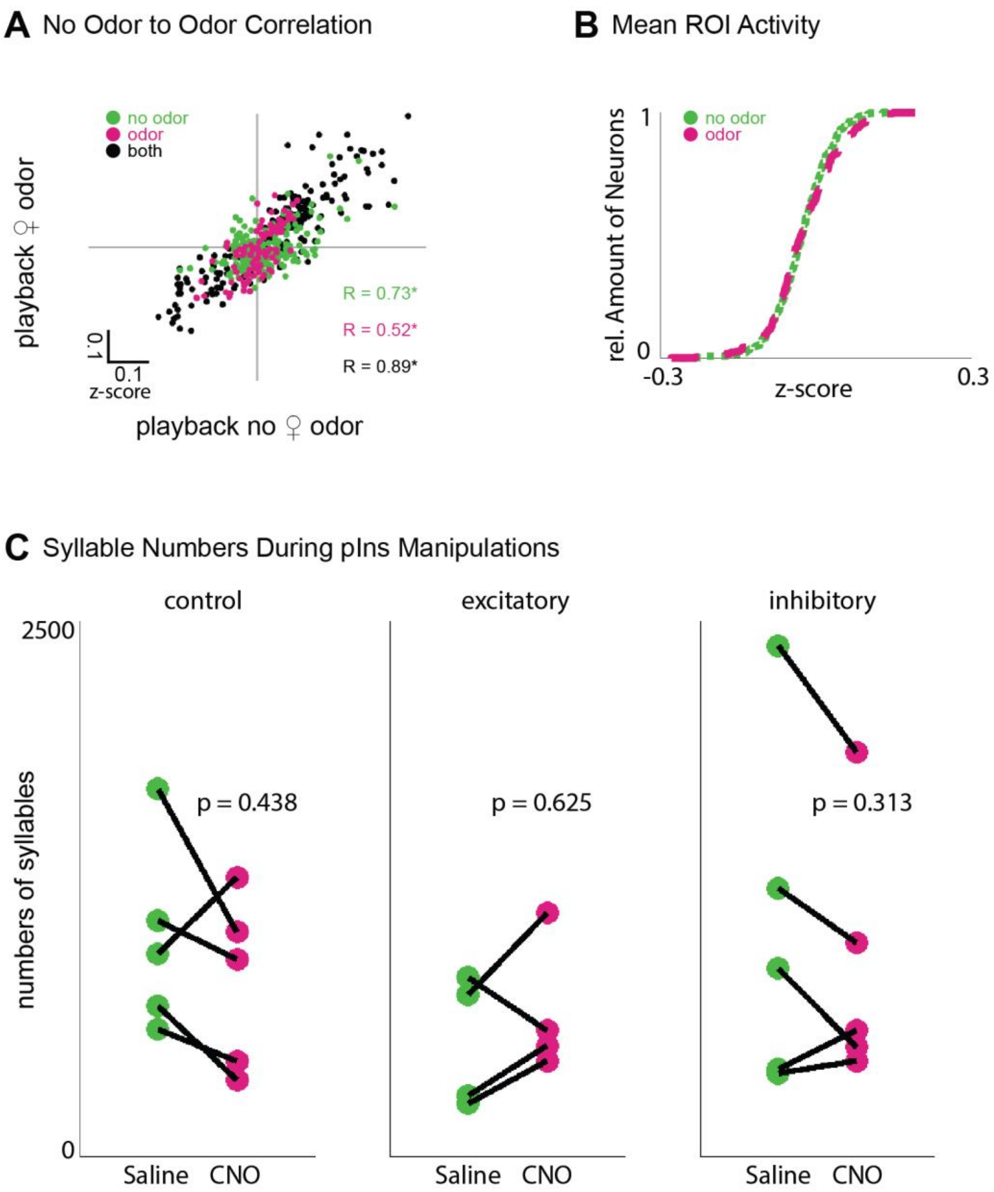

Supplemental figure 6: Odor does not directly modulate baseline activity of pIns ROIs. (A) Pearson correlation ($p < 0.05$) of mean activities of ROIs responsive during USV playback under the 2p setup (N = 4 males). Colors depict responsiveness in neutral airflow (green), positive airflow (magenta) and in both (black). (B) Cumulative distribution function (Kolmogorov-Smirnoff, $p > 0.05$) of baseline activity during neutral airflow (green) and positive airflow (magenta). (C) Syllable numbers of males uttered during courtship for control (left), excitatory (middle), and inhibitory (right) when treated with saline (green) and CNO (magenta). P-values show no significant changes (Wilcoxon). Each dot represents one recording session of one mouse.

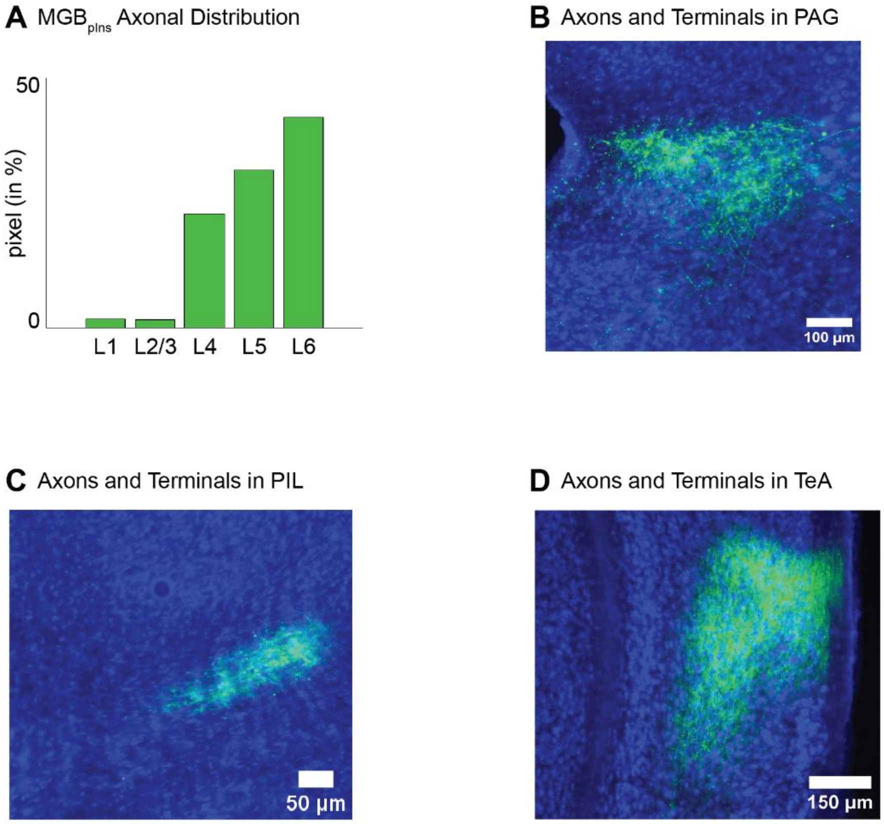

Supplemental Figure 7: The auditory thalamus (MGB) has strong axonal projections to L4-6 of the posterior insula (pIns) and efferent labeling reveals varicosities in fields of axon terminals. (A) Distribution plot of axonal projections of MGB_{pIns} neurons in percentage of pixels. (B-D) Selected examples showing axon terminal fields of pIns efferents (scale bar (B): 100 µm; scale bar (C): 50 µm; scale bar (D): 150 µm). Abbreviations: MGB, auditory thalamus; L1, cortical layer 1; L2/3, cortical layers 2&3; L4, cortical layer 4; L5, cortical layer 5; L6, cortical layer 6; PAG, periaqueductal grey; PIL, posterior intrathalamic nucleus; TeA, temporal association cortex;
